# Comment on “The effect of COVID-19 vaccination on serum levels of anti-Mullerian hormone in women of reproductive age”

**DOI:** 10.5935/1518-0557.20250054

**Published:** 2025

**Authors:** Hinpetch Daungsupawong, Viroj Wiwanitkit

**Affiliations:** 1 Private Academic Consultant, Phonhong, Laos; 2 Department of Research Analytics, Saveetha Dental College and Hospitals, Saveetha Institute of Medical and Technical Sciences Saveetha University, Chennai, India

Dear Editor, we would like to comment on “The effect of COVID-19 vaccination on serum levels of anti-Mullerian hormone in women of reproductive age” (Oliveira *et al*., 2025). The purpose of this study was to investigate the influence of COVID-19 immunization (AstraZeneca^®^ and CoronaVac^®^) on anti-Müllerian hormone (AMH) levels in high-risk women utilizing a retrospective mixed-group study at FMABC from 2021 to 2022. Statistical analysis was carried out in Stata 14 utilizing tests such as the Friedman and Mann-Whitney U tests to compare AMH levels before and after immunization in different groups. The study found no statistically significant variations in AMH levels following immunization, regardless of age group (<35 or ≥35 years) or COVID-19 exposure status.

In terms of statistics, non-parametric tests may not be acceptable when comparing AMH levels across many measurements, as this may result in ambiguous conclusions. When dealing with atypical data distributions, utilizing a more appropriate technique for the data features, such as a mixed effects model, can help to make the analysis more precise and comprehensive. In the future, investigations may focus on increasing the sample size and taking into account factors that may influence AMH levels, such as evaluating the effects of other vaccines or employing methodologies to examine the long-term effects of vaccines on women’s fertility. Furthermore, utilizing a technology with high resolution to test hormone levels may help to obtain more precise data, which can be used for medical decision making.

Although the findings of this study did not show statistically significant differences in AMH levels, there are some limitations to the research design and statistical methods, such as the small sample size (38 people), which may not accurately reflect the general population, preventing the conclusions from being fully generalized. Furthermore, failure to account for the possible effects of other factors, such as exercise levels or hormone use, may be a source of concern in future research.
